# Sulfatase-1 overexpression indicates poor prognosis in urothelial carcinoma of the urinary bladder and upper tract

**DOI:** 10.18632/oncotarget.17590

**Published:** 2017-05-03

**Authors:** Hsiang-Ying Lee, Bi-Wen Yeh, Ti-Chun Chan, Kei-Fu Yang, Wei-Ming Li, Chun-Nung Huang, Hung-Lung Ke, Ching-Chia Li, Hsin-Chih Yeh, Peir-In Liang, Yow-Ling Shiue, Wen-Jeng Wu, Chien-Feng Li

**Affiliations:** ^1^ Graduate Institute of Clinical Medicine, College of Medicine, Kaohsiung Medical University, Kaohsiung, Taiwan; ^2^ Graduate Institute of Medicine, College of Medicine, Kaohsiung Medical University, Kaohsiung, Taiwan; ^3^ Department of Urology, Kaohsiung Medical University Hospital, Kaohsiung, Taiwan; ^4^ Department of Urology, School of Medicine, College of Medicine, Kaohsiung Medical University, Kaohsiung, Taiwan; ^5^ Department of Pathology, Chi-Mei Medical Center, Tainan, Taiwan; ^6^ Institute of Biomedical Sciences, National Sun Yat-Sen University, Kaohsiung, Taiwan; ^7^ Department of Urology, Ministry of Health and Welfare Pingtung Hospital, Pingtung, Taiwan; ^8^ Department of Urology, Kaohsiung Municipal Ta-Tung Hospital, Kaohsiung, Taiwan; ^9^ Department of Pathology, Kaohsiung Medical University Hospital, Kaohsiung, Taiwan; ^10^ Center for Infectious Disease and Cancer Research, Kaohsiung Medical University, Kaohsiung, Taiwan; ^11^ Center for Stem Cell Research, Kaohsiung Medical University, Kaohsiung, Taiwan; ^12^ Institute of Medical Science and Technology, National Sun Yat-Sen University, Kaohsiung, Taiwan; ^13^ Department of Biotechnology, Southern Taiwan University of Science and Technology, Tainan, Taiwan; ^14^ National Cancer Research Institute, National Health Research Institutes, Tainan, Taiwan; ^15^ Department of Internal Medicine and Cancer Center, Kaohsiung Medical University Hospital, Kaohsiung Medical University, Kaohsiung, Taiwan

**Keywords:** urothelial carcinoma, transcriptome, SULF1, prognosis

## Abstract

Urothelial carcinoma (UC), arising from the urothelium of the urinary tract, can occur in the upper (UTUC) and the urinary bladder (UBUC). A representative molecular aberration for UC characteristics and prognosis remains unclear. Data mining of Gene Expression Omnibus focusing on UBUC, we identified sulfatase-1 (*SULF1*) upregulation is associated with UC progression. SULF1 controls the sulfation status of heparan sulfate proteoglycans and plays a role in tumor growth and metastasis, while its role is unexplored in UC. To first elucidate the clinical significance of *SULF1* transcript expression, real-time quantitative RT-PCR was performed in a pilot study of 24 UTUC and 24 UBUC fresh samples. We identified that increased *SULF1* transcript abundance was associated with higher primary tumor (pT) status. By testing SULF1 immunoexpression in independent UTUC and UBUC cohorts consisted of 340 and 295 cases, respectively, high SULF1 expression was significantly associated with advanced pT and nodal status, higher histological grade and presence of vascular invasion in both UTUC and UBUC. In multivariate survival analyses, high SULF1 expression was independently associated with worse DSS (UTUC hazard ratio [HR] = 3.574, *P* < 0.001; UBUC HR = 2.523, *P* = 0.011) and MeFS (UTUC HR = 3.233, *P* < 0.001; UBUC HR = 1.851, *P* = 0.021). Furthermore, depletion of SULF1 expression by using RNA interference leaded to impaired cell proliferative, migratory, and invasive abilities *in vitro*. In addition, we further confirmed oncogenic role of SULF1 with gain-of function experiments. In conclusion, our findings implicate the oncogenic role of SULF1 expression in UC, suggesting SULF1 as a prognostic and therapeutic target of UC.

## INTRODUCTION

Urothelial carcinomas (UCs) arising from the transitional epithelium of the urinary tract are the fourth most common tumors after prostate (or breast), lung and colorectal cancers [1]. These carcinomas grow in the upper urinary tract (pyelocaliceal cavities and ureter) or lower urinary tract (bladder and urethra). Bladder cancer is the most common malignancy of the urinary tract and accounts for 90–95% of UCs [2]. Upper tract urothelial carcinomas (UTUCs) are relatively rare compared with urinary bladder UCs (UBUCs) in Western society [3]. The male-to-female ratio of UTUC is approximately 2-3:1, and pyelocaliceal tumors are approximately two to three times as common as ureteral tumors [4]. However, UTUC accounts for up to 30% of all UCs in Taiwan, and the incidence is approximately equal in men and women, similar to renal pelvis and ureter events [5]. UCs are characterized by frequent recurrence, and even with early diagnosis, poor clinical outcomes were reported with progression into advanced or metastatic disease [6]. Currently, no biomarkers can fulfill the clinical and statistical criteria for better cancer detection, outcome prediction, treatment decision-making or therapy monitoring. Therefore, identification of novel prognostic molecular markers for UC development and progression is needed.

Heparan sulfate proteoglycans (HSPGs) play essential roles in various biological processes and organ systems. These substances are located on the surfaces of most animal cells and represent a crucial element of the extracellular matrix (ECM). HSPGs are composed of a restricted set of core proteins to which are covalently linked one or more heparan sulfate (HS) glycosaminoglycan (GAG) chains. The great diversity of HS structures includes the length and size of the sulfated and non-sulfated regions as well as disaccharide composition [7–8]. The variations differ in specific ways and are dynamically managed at distinct cell type and tissue levels, during development, and in pathological statuses such as cancer progression. These markers manifest the primary principle dictating that HS-ligand interactions are largely dependent on specific sulfation patterns in segments of the chain with distinct docking sites for the various ligands [9–10].

Human sulfatase-1 and sulfatase-2 (SULF1 and SULF2, hereafter referred to as the SULFs) are novel enzymes discovered in the early 2000s that control the sulfation status of HSPGs and demonstrate up- or down-regulation in different types of malignancies to promote or repress tumor growth and metastasis [11]. According to the previous literature, SULF1 and SULF2 may have opposing effects in cancer progression despite similar structures and activities. Earlier evidence has shown that SULF1 downregulates multiple signaling pathways via HS-binding growth factors such as FGF2, HGF, HB-EGF, VEGF, PDGF and amphiregulin. In this respect, SULF1 displayed tumor suppressor function in hepatocellular carcinoma (HCC), ovarian cancer, kidney cancer and multiple myeloma [12]. However, more recent studies revealed over-expressed SULF1 in cancers such as pancreatic, gastric, and lung adenocarcinoma, glioma, invasive breast carcinoma, and leukemia, which exhibited protumorigenic effects [13–16]. Overall, these divergent results highlight a poor understanding of the complicated mechanisms and multifactorial implications of the SULF1 in cancers.

The role of SULF1 in UCs is limited according to known information. We conducted this study to evaluate the expression status of SULF1 associated with disease states in human UCs. Furthermore, we assessed the effects of SULF1 on patient survival and identified that the expression of SULF1 is a prognostic biomarker in UCs.

## RESULTS

### *SULF1* identified as a significant differentially upregulated gene implicated in tumor progression in UBUC

**Figure 1:**
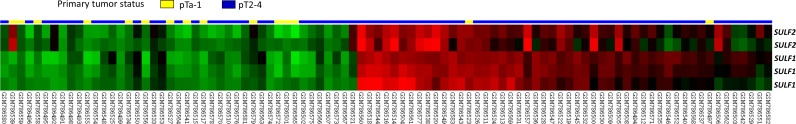
Reappraisal of transcriptome dataset in urothelial carcinoma (GSE31684) Clustering analysis of genes focusing on those involving heparan sulfate proteoglycan metabolic process revealed *SULF1* is the most significantly up-regulated gene associated with increments of pT status, followed by *SULF2*, prompting us to further validate their significance. Tissue specimens from tumors with different pT statuses are indicated on top of the heatmap, and expression levels of up-regulated and down-regulated genes are represented as a spectrum of brightness or red and green, respectively. Cases unaltered in mRNA transcriptional level are coded black.

Using data mining from published transcriptomic datasets of UBUCs (GSE31684 and GSE32894), *SULF1* and *SULF2* were identified as significant genes showing upregulation during tumor progression among those associated with the heparan sulfate proteoglycan metabolic process (GO:0030201) (Figure 1, Supplementary Figure 1 and Table 1, Supplementary Table 1). Of these *SULF1* showed a log2 ratio of 2.8929 and 1.4253-fold upregulation and *SULF2* showed 1.7641 and 0.7979-fold upregulation in GSE31684 and GSE32894, respectively.

**Table 1:** Summary of differentially expressed genes associated with heparan sulfate proteoglycan metabolic process in the transcriptome of urothelial carcinoma of urinary bladder (GSE31684)

Probe	Comparing T2-4 to Ta-T1		Gene Symbol	Biological Process	Molecular Function
	log ratio	*p*-value			
212353_at	2.8929	0	*SULF1*	apoptosis, heparan sulfate proteoglycan metabolic process, metabolic process	arylsulfatase activity, calcium ion binding, hydrolase activity, metal ion binding, sulfuric ester hydrolase activity
212354_at	2.2812	0	*SULF1*	apoptosis, heparan sulfate proteoglycan metabolic process, metabolic process	arylsulfatase activity, calcium ion binding, hydrolase activity, metal ion binding, sulfuric ester hydrolase activity
224724_at	1.7641	0	*SULF2*	heparan sulfate proteoglycan metabolic process, metabolic process	arylsulfatase activity, calcium ion binding, hydrolase activity, metal ion binding, sulfuric ester hydrolase activity
212344_at	1.5038	0.0036	*SULF1*	apoptosis, heparan sulfate proteoglycan metabolic process, metabolic process	arylsulfatase activity, calcium ion binding, hydrolase activity, metal ion binding, sulfuric ester hydrolase activity
233555_s_at	0.9063	0.001	*SULF2*	heparan sulfate proteoglycan metabolic process, metabolic process	arylsulfatase activity, calcium ion binding, hydrolase activity, metal ion binding, sulfuric ester hydrolase activity

### *SULF1* but not *SULF2* transcript expression predicts survival in UBUC transcriptomic dataset

**Figure 2:**
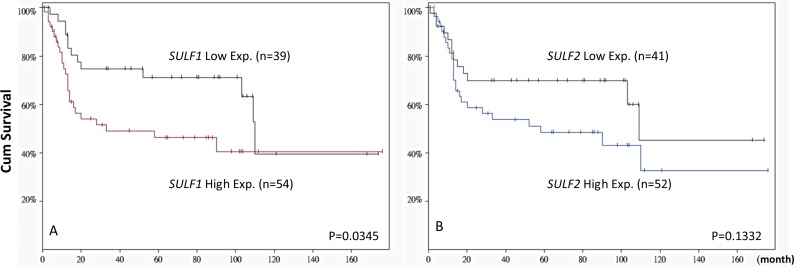
Patient outcome stratified by SULF1 and SULF2 transcript levels based on GSE31684 The expression level of *SULF1* significantly predicts worse patient outcome (*left*, *P* = 0.0345). While *SULF2* expression status is not predictive for patient survival (right, *P* = 0.1332).

Subdividing 93 cases from GSE31684 into *SULF1* high-expression (*n* = 54) and low-expression (*n* = 39) clusters showed that a high expression level of *SULF1* significantly determined worse patient survival (*P* = 0.0345, Figure 2). However, high *SULF2* expression (*n* = 52) did not significantly predict patient outcome (*P* = 0.1332). The findings prompt us to further characterize the significance of SULF1 transcript and protein expression in our large cohort of urothelial carcinoma.

### Higher *SULF1* mRNA expression is associated with advanced pT stages in both UTUC and UBUC

**Figure 3:**
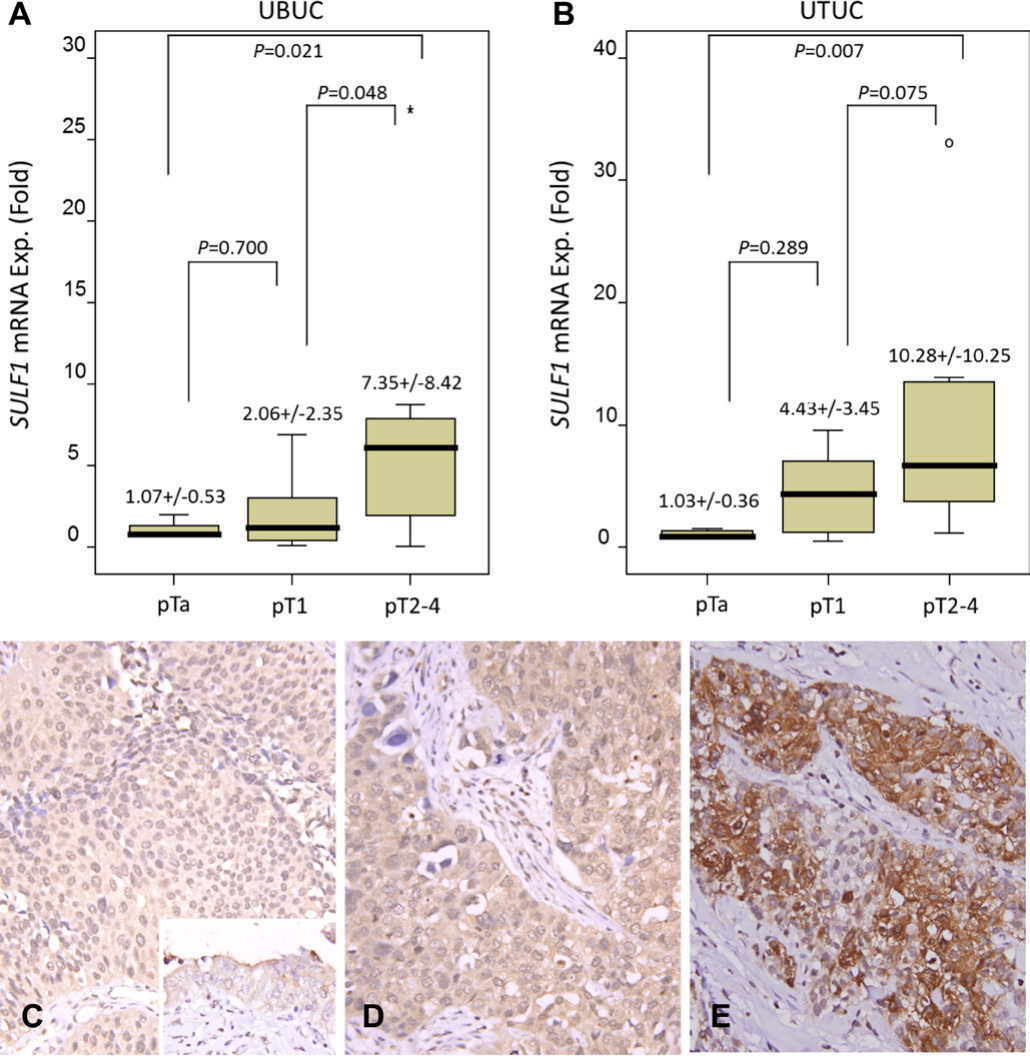
Validation of SULF1 transcript and protein expression *SULF1* mRNA level was significantly increased in both UBUCs (**A**) and UTUCs (**B**) with advanced primary pT status. (*p* = 0.021 and *p* = 0.007, respectively). Immunhistochemically, SULF1 is barely detected in non-tumorous urothelium (C, inset) and non-invasive UC (**C**) and shows a mild increase in superficially invasive UC (**D**). The representative high-grade and high-stage UC shows a bright SULF1 immunoreactivity (**E**).

The *SULF1* mRNA expression was analyzed in both UTUC and UBUC samples. It showed *SULF1* mRNA expression significantly increased in higher stage tumors in both UTUC (*P* = 0.007) and UBUC (*P* = 0.021), verifying the important role of SULF1 in cancer progression (Figure 3A, 3B).

### Clinicopathological findings of UTUC

The clinicopathological characteristics of the UTUC patients are listed in Table 2. Between low and high SULF1 expression, gender has no significant difference and the age of patients at diagnosis ranged from 34 to 87 years (median 68). Sixty-two patients (18.2%) had multiple foci tumors, and 49 (14.4%) had tumors in both the renal pelvis and ureter simultaneously. Most patients (*n* = 284, 83.5%) were high histological grade and advanced pT stages (pT2-T4) were noted in 159 (46.8%) of cases. Around half (*n* = 167, 49.1%) of the cases showed frequent mitosis. 106 cases (31.2%) and 19 cases (5.9%) presented vascular invasion and perineural invasion, respectively. Nodal metastasis was noted in 28 patients (8.2%).

**Table 2:** Correlations between SULF1 expression and other important clinicopathological parameters in urothelial carcinomas

Parameter	Category	Upper Urinary Tract Urothelial Carcinoma				Urinary Bladder Urothelial Carcinoma			
		Case No.	SULF1 Expression		*p*–value	Case No.	SULF1 Expression		*p*–value
			Low	High			Low	High	
Gender	Male	158	84	74	0.277	216	105	111	0.489
	Female	182	86	96		79	42	37	
Age (years)	< 65	138	75	63	0.185	121	50	71	0.015*
	≥ 65	202	95	107		174	97	77	
Tumor location	Renal pelvis	141	64	77	0.344	–	–	–	–
	Ureter	150	79	71		–	–	–	–
	Renal pelvis & ureter	49	27	22		–	–	–	–
Multifocality	Single	278	138	140	0.779	–	–	–	–
	Multifocal	62	32	30		–	–	–	–
Primary tumor (T)	Ta	89	60	29	< 0.001*	84	57	27	< 0.001*
	T1	92	47	45		88	54	34	
	T2-T4	159	63	96		123	36	87	
Nodal metastasis	Negative (N0)	312	164	148	0.002*	266	142	124	< 0.001*
	Positive (N1–N2)	28	6	22		29	5	24	
Histological grade	Low grade	56	36	20	0.019*	56	37	19	0.007*
	High grade	284	134	150		239	110	129	
Vascular invasion	Absent	234	133	101	< 0.001*	246	134	112	< 0.001*
	Present	106	37	69		49	13	36	
Perineural invasion	Absent	321	162	159	0.479	275	141	134	0.066
	Present	19	8	11		20	6	14	
Mitotic rate (per 10 high power fields)	< 10	173	94	79	0.104	139	76	63	0.116
	> = 10	167	76	91		156	71	85	

*Statistically significant.

### Clinicopathological findings of UBUC

Of the 295 UBUC cases, male (*n* = 216, 73.2%) is more than female. As shown in Table 2, 239 (81%) patients had high histological grades, and 123 cases (41.7%) were at a muscle-invasion stage (pT2-T4) during diagnosis. High mitotic activity (≥ 10) and lymph node metastasis were found in 156 cases (52.9%) and 29 cases (23.6%), respectively. In addition, 49 cases (16.6%) have vascular invasion and perineural invasion were present in 20 cases (6.8%).

### Correlations of immunoreactivity of SULF1 and SULF2 with clinicopathological features of UTUC and UBUC

SULF1 and SULF2 show variable cytoplasmic expression in both UTUC and UBUC. The tumors were dichotomized into those with low and high SULF1 expression, as demonstrated in Figure 3C–3E, Table 2, high SULF1 expression was significantly associated with age less than 65 (UBUC, *P* = 0.015), more advanced primary tumor pT stage (*P* < 0.001, both UTUC and UBUC), lymph node metastasis (UTUC, *P* = 0.002; UBUC, *P* < 0.001), higher histological grade (UTUC, *P* = 0.019; UBUC, *P* = 0.007) and vascular invasion (*P* < 0.001, both UTUC and UBUC) in urothelial carcinomas. As showed in Supplementary Figure 2, high-grade and high-stage UC shows a bright SULF2 immunoreactivity. The clinicopathological associations of SULF2 expression in UTUC and UBUC patients are listed in Supplementary Table 2. Renal pelvis location, single site tumor, advanced pT stages, lymph node metastasis, high grade, vascular invasion, higher mitotic rate present high SULF2 expression in UTUC significantly. Advanced pT stages, lymph node metastasis, high grade present high SULF2 expression in UBUC significantly.

### Survival analysis for UTUC

**Figure 4:**
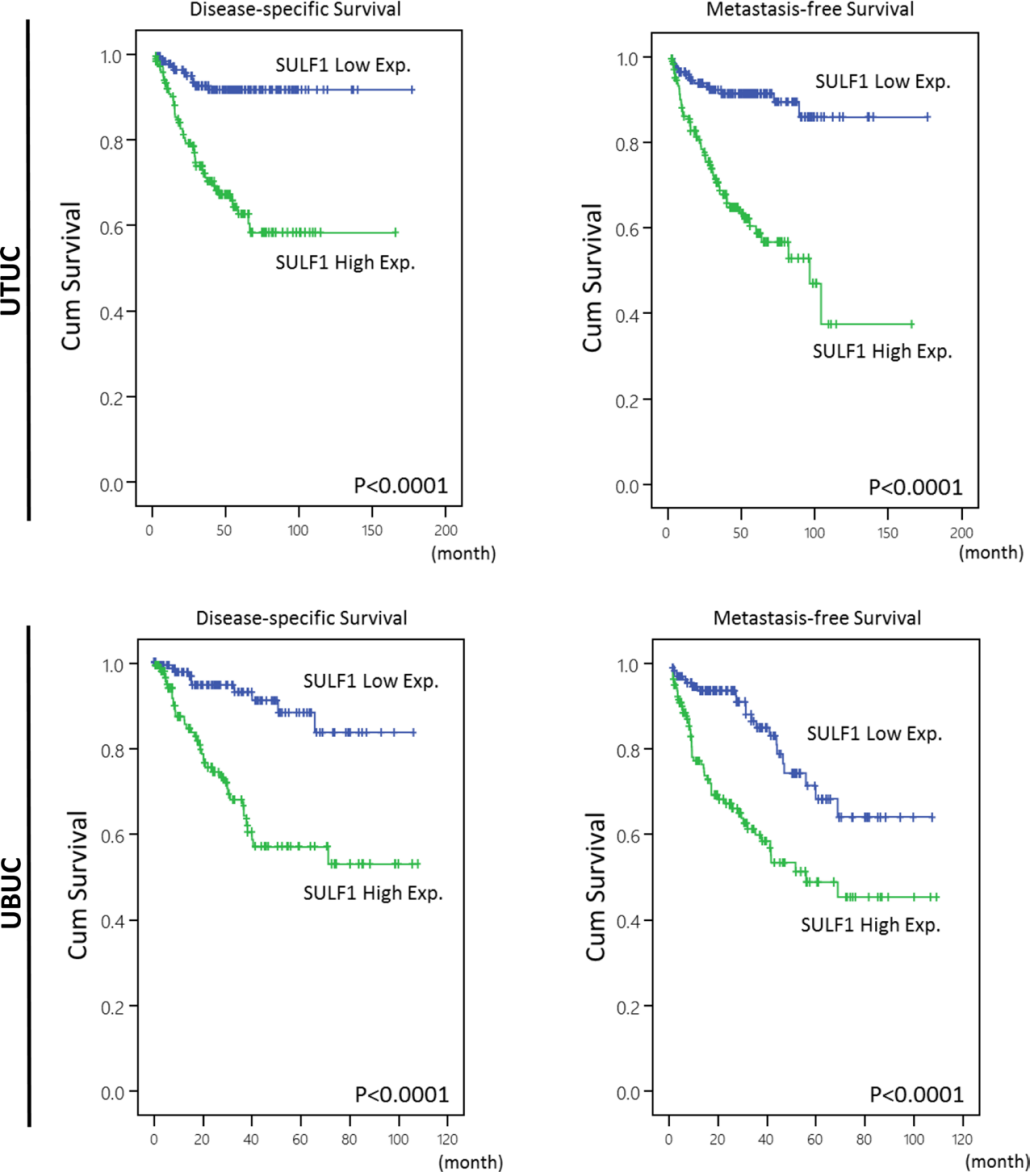
Kaplan-Meier plots of disease-specific survival (DSS) and metastasis-free survival (MeFS) of UTUCs and UBUCs SULF1 expression significantly predicts inferior DSS and MeFS in UTUC and UBUC (all *P* < 0.0001).

Table 3 showed univariate and multivariate analyses of the association between clinical outcomes and various clinicopathological features of UTUC cases. In univariate and multivariate analysis, high SULF1 immunoexpression together with a number of important clinicopathological factors including tumor multifocality, higher primary tumor pT stage status, nodal status, high histological grade, and perineurial invasions were predictive of worse outcomes in terms of disease-specific survival (DSS). After univariate and multivariate analysis in metastasis-free survival (MeFS), high SULF1 expression, tumor multifocality, nodal metastasis, high histological grade, vascular and perineural invasion remained as independently significant prognosticators. From Kaplan-Meier analysis, high expression of SULF1 also predict significantly worse DSS (Hazard Ratio [H.R.] = 3.574, *P* < 0.001) and MeFS (H.R. = 3.233, *P* < 0.001) in UTUC (Figure 4, upper panel). As shown in Supplementary Table 3 and Supplementary Figure 3, high expression of SULF2 predicts significantly worse MeFS (*P* = 0.026) but not DSS and is not enrolled into multivariate survival analysis.

**Table 3:** Univariate log-rank and multivariate analyses for disease-specific and metastasis-free survivals in upper urinary tract urothelial carcinoma

Parameter	Category	Case No.	Disease-specific Survival					Metastasis–free Survival				
			Univariate analysis		Multivariate analysis			Univariate analysis		Multivariate analysis		
			No. of event	*p*-value	R.R.	95% C.I.	*p*–value	No. of event	*p*–value	R.R.	95% C.I.	*p*–value
Gender	Male	158	28	0.8286	–	–	–	32	0.7904	–	–	–
	Female	182	33		–	–	–	38		–	–	–
Age (years)	< 65	138	26	0.9943	–	–	–	30	0.8470	–	–	–
	≥ 65	202	35		–	–	–	40		–	–	–
Tumor side	Right	177	34	0.7366	–	–	–	38	0.3074	–	–	–
	Left	154	26		–	–	–	32		–	–	–
	Bilateral	9	1		–	–	–	0		–	–	–
Tumor location	Renal pelvis	141	24	0.0079*	1	–	0.536	31	0.0659	–	–	–
	Ureter	150	22		0.873	0.472–1.615		25		–	–	–
	Renal pelvis & ureter	49	15		2.139	0.584–7.837		14		–	–	–
Multifocality	Single	273	48	0.0026*	1	–	0.009*	52	0.0127*	1	–	0.002*
	Multifocal	62	18		2.734	1.290–5.795		18		2.452	1.407–4.308	
Primary tumor (T)	Ta	89	2	< 0.0001*	1	–	0.015*	4	< 0.0001*	1	–	0.250
	T1	92	9		2.388	0.501–11.386		15		2.099	0.677–6.511	
	T2–T4	159	50		4.935	1.094–22.250		51		2.216	0.693–7.088	
Nodal metastasis	Negative (N0)	312	42	< 0.0001*	1	–	< 0.001*	55	< 0.0001*	1	–	0.001*
	Positive (N1–N2)	28	19		5.155	2.757–9.639		15		2.795	1.501–5.203	
Histological grade	Low grade	56	4	0.0215*	1	–	0.024*	3	0.0027*	1	–	0.019*
	High grade	284	57		3.658	1.184–11.298		67		4.345	1.267–14.901	
Vascular invasion	Absent	234	24	< 0.0001*	1	–	0.344	26	< 0.0001*	1	–	0.008*
	Present	106	37		1.353	0.724–2.529		44		2.362	1.252–4.457	
Perineural invasion	Absent	321	50	< 0.0001*	1	–	< 0.001*	61	< 0.0001*	1	–	0.005*
	Present	19	11		4.297	2.038–9.059		9		2.930	1.381–6.219	
Mitotic rate (per 10 high power fields)	< 10	173	27	0.167	–	–	–	30	0.0823	–	–	–
	> = 10	167	34		–	–	–	40		–	–	–
SULF1 expression	Low	170	11	< 0.0001*	1	–	< 0.001*	14	< 0.0001*	1	–	< 0.001*
	High	170	50		3.574	1.818–7.027		56		3.233	1.773–5.895	

* Statistically significant.

### Survival analysis for UBUC

After analyzing in UBUC, a list of clinicopathological variables significantly predicted worse DSS and MeFS, including advanced pT stage and nodal status, higher histological grade, the presence of vascular and perineurial invasions and higher mitotic activity in univariate analysis, shown in Table 4. Of note, high SULF1 expression not only significantly predicted worse outcome in both univariate and multivariate analysis but also significantly impacted DSS (H.R. = 2.523, *P* = 0.011) and MeFS (H.R. = 1.851, *P* = 0.021) in Kaplan-Meier analysis (Figure 4, lower panel). SULF2 expression is not predictive for worse patient outcome.

**Table 4:** Univariate log-rank and multivariate analyses for disease-specific and metastasis-free survivals in urinary bladder urothelial carcinoma

Parameter	Category	Case No.	Disease-specific Survival					Metastasis–free Survival				
			Univariate analysis		Multivariate analysis			Univariate analysis		Multivariate analysis		
			No. of event	*p*–value	R.R.	95% C.I.	*p*–value	No. of event	*p*–value	R.R.	95% C.I.	*p*–value
Gender	Male	216	41	0.4446	–	–	–	60	0.2720	–	–	–
	Female	79	11		–	–	–	16		–	–	–
Age (years)	< 65	121	17	0.1136	–	–	–	31	0.6875	–	–	–
	≥ 65	174	35		–	–	–	45		–	–	–
Primary tumor (T)	Ta	84	1	< 0.0001*	1	–	0.001*	4	< 0.0001*	1	–	0.012*
	T1	88	9		6.192	0.672–57.068		23		5.076	1.476–17.454	
	T2–T4	123	42		20.271	2.267–181.274		49		6.639	1.911–23.057	
Nodal metastasis	Negative (N0)	266	41	0.0002*	1	–	0.489	61	< 0.0001*	1	–	0.068
	Positive (N1–N2)	29	11		1.281	0.635–2.586		15		1.768	0.959–3.260	
Histological grade	Low grade	56	2	0.0013*	1	–	0.921	5	0.0007*	1	–	0.675
	High grade	239	50		1.081	0.230–5.082		71		1.250	0.440–3.550	
Vascular invasion	Absent	246	37	0.0024*	1	–	0.166	54	0.0001*	1	–	0.827
	Present	49	15		0.618	0.313–1.221		22		0.937	0.522–1.682	
Perineural invasion	Absent	275	44	0.0001*	1	–	0.081	66	0.0007*	1	–	0.235
	Present	20	8		2.90	0.914–4.778		10		1.561	0.748–3.256	
Mitotic rate (per 10 high power fields)	< 10	139	12	< 0.0001*	1	–	0.014*	23	< 0.0001*	1	–	0.011*
	> = 10	156	40		2.313	1.182–4.526		53		1.952	1.164–3.273	
SULF1 expression	Low	147	10	< 0.0001*	1	–	0.011*	22	< 0.0001*	1	–	0.021*
	High	148	42		2.523	1.233–5.165		54		1.851	1.098–3.119	

* Statistically significant.

### SULF1 promotes *in vitro* aggressiveness of UC cells

**Figure 5:**
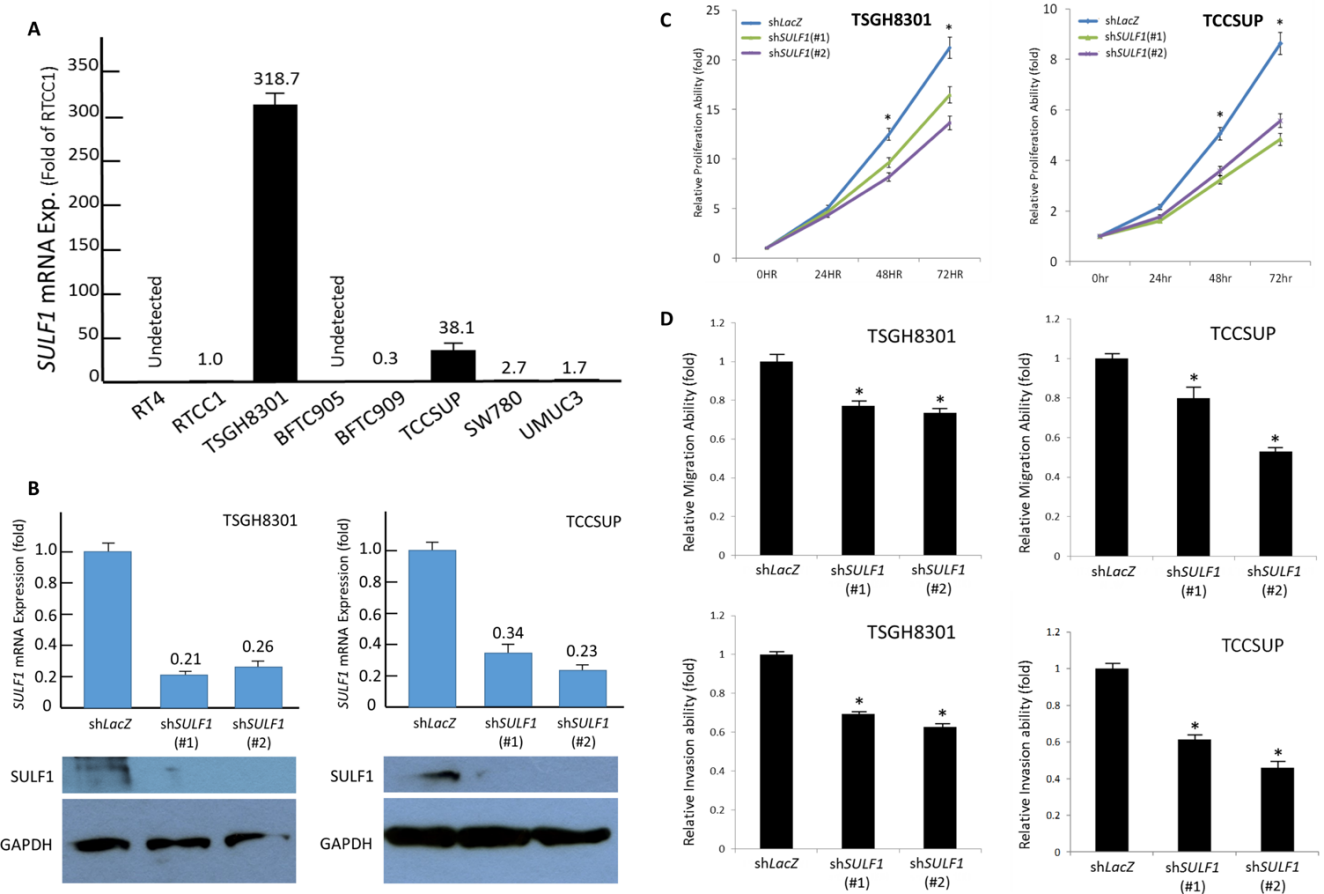
SULF1 expression promotes growth of UC cells *in vitro* TSGH8301 and TCCSUP cell lines reveal the most abundant *SULF1* transcript level (**A**). These two cell lines with high endogenous SULF1 expression are stably silenced against SULF1 expression by a lentiviral vector bearing one of the two clones of SULF1 short hairpin (sh)RNA with different sequences for both TSGH8301 and TCCSUP cells. The efficiency of RNA silencing is confirmed by both quantitative RT-PCR (*upper*) and western blotting (*lower*) assays (**B**). Using 2,3-bis-(2-methoxy-4-nitro-5-sulfophenyl)-2H-tetrazolium-5-carboxanilide (XTT), we confirmed depletion of SULF1 impairs cell growth (**C**) as well as cell migration (**D**, *upper*) and invasion abilities (**D**, *lower*).(*P < 0.05).

The above-mentioned results prompted us to further work on the biological significances of SULF1 expression UC. We first evaluated the endogenous *SULF1* expression by using real-time quantitative RT-PCR in a list of UC cell lines and identified TSGH8301 and TCCSUP have most abundant *SULF1* transcript level. To further clarify whether SULF1 modulates cell aggressiveness, we performed SULF1 knockdown by means of short hairpin RNA to deplete SULF1 expression in TSGH8301 and TCCSUP cells. Evaluated by (2,3-bis-(2-methoxy-4-nitro-5-sulfophenyl)-2H-tetrazolium-5-carboxanilide) XTT, modified Boyden chamber migration and invasion assays, we identified depletion of SULF1 leaded to significant diminished cancer cell proliferation, migration, and invasion. These findings demonstrated that SULF1 promotes *in vitro* aggressiveness of UC cells (Figure 5).

### SULF1 expression promotes *in vitro* proliferation of UC cells

**Figure 6:**
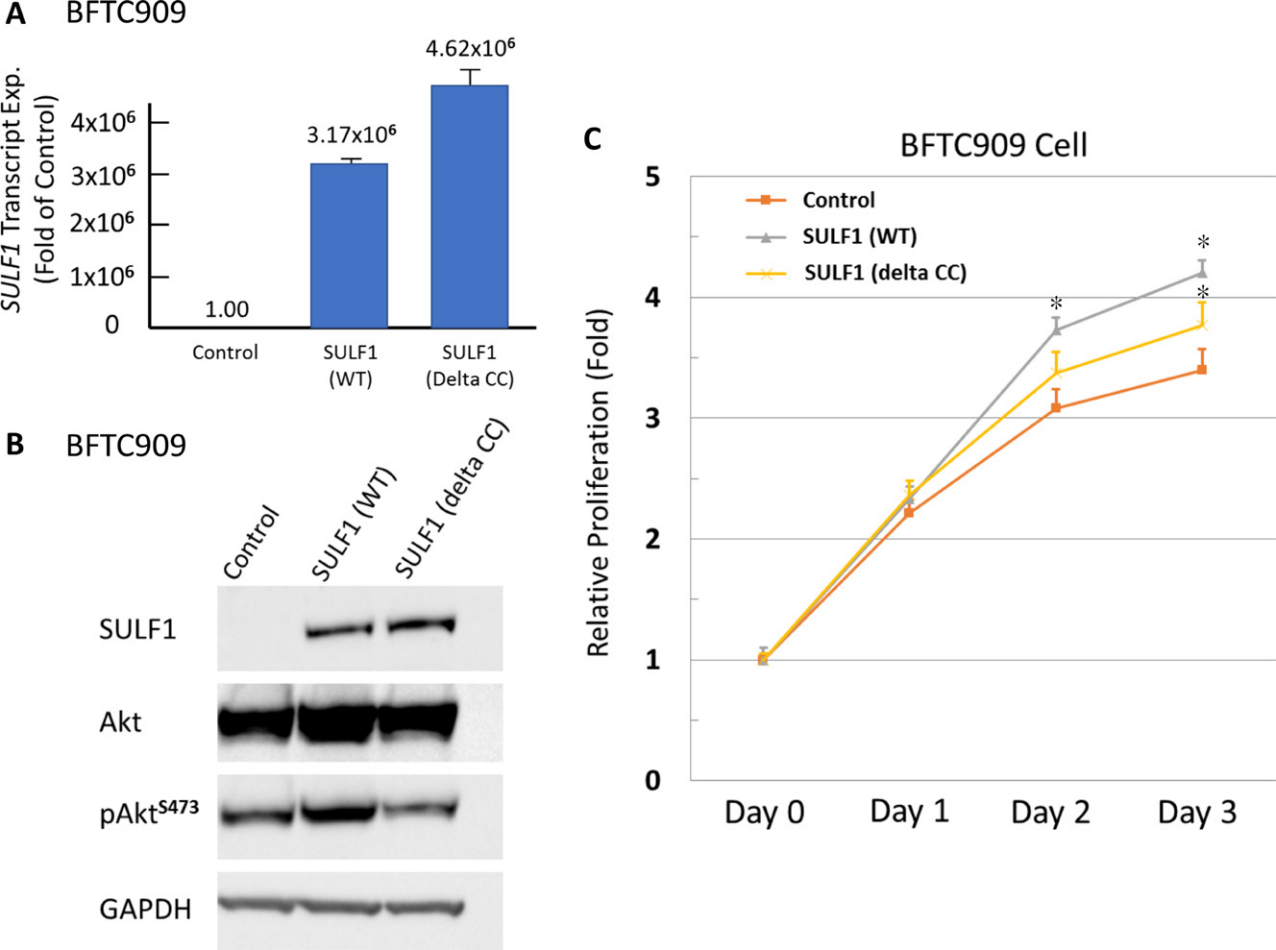
Exogenous SULF1 expression promotes UC cells proliferation *in vitro* Exogenous expression of wild-type and mutant (delta CC) SULF1 has been performed in SULF1-low-expressing BFTC909 cell line. The efficiency is confirmed by both quantitative RT-PCR (**A**) and western blotting assays (**B**). The significant increased phosphorylated Akt can be detected in that with wild-type SULF1, while not in that with delta CC mutation, suggesting the activation of Akt pathway is associated with enzymatic function of SULF1. Exogenous expression of wild-type SULF1 significantly promotes cell proliferation (**C**). That with delta CC mutated SULF1 revealed a proliferation rate between control cells and that with wild-type SULF1, indicating the oncogenic role of SULF1 may be partly relay on its enzymatic function.

It revealed a low SULF1 transcript expression in BFTC909 cell lines (Figure 5A) which is eligible for gain-of function experiments by using wild-type (WT) and mutant (delta CC, with impaired enzymatic function) SULF1 (Figure 6A). We identified SULF1 (WT) significantly promoted cell proliferation while the effect is significantly diminished but not completely lost by a delta CC mutation (Figure 6B, 6C). The western blotting showed Akt phosphorylation increased by exogenous wild-type SULF1 expression but not in that with delta CC mutation, suggesting a role of SULF1 enzymatic function in activating Akt pathway to promote it oncogenic nature. Of interest, SULF1 also leaded to significantly increased cancer cell migration and invasion, which were not depleted by delta CC mutation (Supplementary Figure 4). These findings not only confirmed the oncogenic role of SULF1 in promoting cell proliferation, migration, and invasiveness, but also first disclosed the oncogenic role of SULF1 might be related to but not totally relay on its enzymatic function.

## DISCUSSION

UC presents either a noninvasive or invasive pattern with different management approaches and prognoses. When a patient with UC progresses to an advanced stage, the survival rate is dramatically decreased if standard treatment is received. A study revealed that the 5-year survival rate in non-muscle invasive UC (pTa, pTis and pT1 stages) was greater than 90% but decreased to less than 50% for pT3 stage and to 5% for pT4 stage [17]. For early identification of patients with potential for advanced status, we must find significant prognostic factors and therapeutic markers for UC and design individualized plans of treatment and surveillance. Modern technologies have been used to identify a variety of molecular markers for this purpose.

Experiments illustrated the crucial characters of HS chains from global HS deficiency in mice that resulted in extraordinary gastrulation leading to embryonic death [7]. Because HSPGs have the ability to bind a large diversity of ligands (namely, cytokines, chemokines, morphogens, enzymes, receptors, growth factors, matrix/adhesion molecules, and plasma proteins), they modulate nutritional metabolism, organize basement membrane barriers, regulate cell signaling and morphogenesis and are involved in cellular crosstalk as well as participate in injury and repair. In the tumor microenvironment, these modifications are known to significantly influence cancer progression, metastasis and prognosis [18].

A number of studies have reported that biosynthesis of HS influenced cell transformation and evolution through the different stages of cancers, including desulfation of 6-O-sulfate [7, 19]. Many researchers also demonstrated the ability of SULF1 and SULF2 in editing post-synthetic sulfation status of 6-O-S from HSPGs, and thus the implication and potential roles of SULFs in malignancies were extensively investigated [20, 21]. However, the precise functions of SULFs in regulatory mechanisms and molecular interactions in a variety of tumors remain unclear. Our study depicted the oncogenic character of SULF1 in UC progression. The major findings of the current study reveal that upregulation of SULFs is found in UCs, and overexpression of SULF1 proteins correlates with worse survival in both UTUC and UBUC patients.

In 2001, Dhoot and colleagues first proved that SULF1 positively regulates the Wnt signaling pathway in embryonic quail (QSULFs). The orthologs and isoforms of QSULF1 were subsequently identified in species including humans (so-called SULF1 and SULF2). Even if the two SULFs are structurally similar, they are unique and markedly different from other members of the sulfatase family. Most of the previously recognized cellular sulfatases cleave sulfate esters from the terminus of the chains (exosulfatases) within the acidic conditions of the lysosomal compartment (intracellular). SULFs localized at the plasma membrane or secreted into the ECM (extracellular) are endoglucosamine-6-sulfatases and show sequence heterology with other sulfatases. These substances selectively catalyze removal of 6-O-sulfate groups, mainly from the S domains of heparan sulfate polymers [22, 23].

Many studies have shown that SULF1 and SULF2 are involved in multiple cellular signaling pathways by modulation of the binding and activating properties of a variety of protein ligands to HS/heparin. Depending on the different SULFs involved, the targeted HS ligands, and the biochemical situations, these pathways are prompted or inhibited and result in pro- or anti-tumor effects. For instance, SULFs have been demonstrated to weaken the affinity of HSPGs for Wnt ligands, which activate signal transduction of Frizzled (Fz) receptors and construct the HS/Wnt/Fz functional compound. This mechanism inducts a series of cytoplasmic reactions that accumulate β-catenin in the nucleus and trigger the Wnt signaling pathway. Via a similar mechanism, BMP4 signaling was enhanced with the action of SULFs via release of an HSPG-binding inhibitor (Noggin) of BMP from the cell surface. GDNF signaling was also reported to be activated by SULFs during mouse neuronal cell protection/regeneration and spermatogenesis [24–26]. By releasing ligands from immobilized HS/heparin (decrease binding ability), SULFs increase the engagement of ligands with receptors and consequently turn on the signaling pathways downstream.

In contrast to upregulation on signaling, SULF1 inhibited the signaling response to several ligands such as HGF, HB-EGF, VEGF, FGF1, FGF2, TGF-β and amphiregulin. Sonic Hedgehog (shh) signaling was enhanced during embryonic development and axonal guidance but repressed in gastric cancer by SULF1. The manifestation may be distinctive depending on different stage of the cancer and the hypoxic level in the tumor microenvironment [27].

FGF2 is a powerful angiogenic factor for HCC. The effects of SULF1 as a tumor suppressor have been identified in HCC for mediating the inhibition of HS-dependent receptor tyrosine kinase signaling in *in vitro* and *in vivo* mouse xenografts. Moreover, knockdown of SULF1 by SULF1-targeting short hairpin RNA (shRNA) constructs significantly attenuated apicidin-induced inhibition of HCC cell migration. Nevertheless, higher expression of SULF1 in HCC tissues at a level 1.5× greater than that of adjacent benign tissues was noted in a third of HCCs. In addition, nearly 40% of patients with high tumor SULF1 expression have the hepatoblast phenotype of HCC, which showed relatively poor survival [28]. The complicated interaction between SULF1 and anti-/pro-tumorigenic signaling molecules signifies its bimodal effect in HCC. It is also interesting that Lai et al. showed a combination of apicidin and doxorubicin leads to an increased DNA-damage and apoptosis in SULF1-expressing HCC cells *in vitro* and invivo [29].

In breast and ovarian cancers, SULF1 exhibition of a tumor suppressive effect was relevant to its ability to attenuate FGF2, HB-EGF, and amphiregulin signaling. According to studies by Liu et al., restoring the expression of SULF1 on ovarian cancer resulted in reduced tumor development and angiogenesis and increased efficacy of anti-cancer agents such as cisplatin [30–31]. SULFs are known to activate Wnt signaling cascade in pancreatic adenocarcinomas, which implies that their higher expression contributes to progression and tumorigenicity of pancreatic cancers. SULF1 mRNA levels in pancreatic cancer samples have been reported as increased compared with normal tissuein these patients, although SULFs were shown to attenuate other pro-tumorigenic signaling pathways and subsequently interfere with cancer advancement, e.g., inhibition of angiogenesis and tumorigenesis by SULF1 *in vivo* [32–33].

Our study showed that high expression of SULF1 was associated with worse prognosis and higher risk of metastasis in both UTUCs and UTUBs. In addition, high SULF1 expression correlates with advanced T-stage, nodal metastasis, higher grade, and vascular invasion, and the results can also be identified from our functional study, not only knockdown of SULF1 expression, but also over-expression SULF1 *in vitro* experiments which demonstrated that SULF1 promotes tumor migration and invasion ability. Interestingly, we identify the phosphorylation of Akt is increased by exogenous wild-type SULF1 but not that with delta CC mutation. Akt is known to fully activated after phosphorylation at two regulatory residues, a threonine residue (p-AktThr308) on the kinase domain and a serine residue (p-AktSer473) on the C-terminal hydrophobic motif. The fully active Akt is known to impact various substrates related to the regulation of cell proliferation and inhibition of apoptosis by phosphorylation downstream substrate so related to cellular survival [34]. Meanwhile, although delta CC mutant SULF1 revealed less pAkt protein, it also can promote cancer cell proliferation, migration and invasion. The phenomenon indicates the oncogenic role of SULF1 may not only from its enzymatic function and may have alternative activating pathways than Akt signaling. However, the exact pathway still needs to be clarified. This trend was also detected in SULF2, although no statistical significance was found. In our results, high SULF2 expression is only associated with worse MeFS in UTUC. Our findings suggest that SULF1 plays a critical role in advanced UCs and shows that SULF1 overexpression has important clinical implications as a prognostic and predictive factor in UCs. Since the imperative role of SULF1 expression was demonstrated from our result, it can considered for clinical application in development of therapy targeting SULF1 or as a biomarker for diagnosis and surveillance strategies. In addition, the SULF1 level may be added to a prognostic model in patients with UCs.

In conclusion, our study found that increased SULF1 expression is significantly predictive of more advanced tumor stage and poorer metastasis-free survival and disease-specific survival in patients with both UTUC and UBUC. These findings demonstrated the oncologic role of SULF1 in UC through SULF1 knockdown and overexpression functional assays. Therefore, SULF1 expression in UCs may be a clinically prognostic factor indicating poor patient survival and a biomarker for development of targeted therapies.

## MATERIALS AND METHODS

### Data mining to identify *SULF1* and *SULF2* transcripts in UC progression

We performed data mining on public domain data from the GEO (Gene Expression Omnibus, National Center Biotechnology information, Bethesda, MD, USA) and identified two datasets, GSE31684 (http://www.ncbi.nlm.nih.gov/geo/query/acc.cgi?acc=GSE31684) (Figure 1) and GSE32894 (http://www.ncbi.nlm.nih.gov/geo/query/acc.cgi?acc=GSE32894) (Supplementary Figure 1), which profiled 93 and 308 UBUCs using Affymetrix U133 Plus 2.0 Array and HumanHT-12 V3.0 expression bead chip, respectively. To analyze the expression level, we imported the raw files into the Nexus Expression 3 statistical software (BioDiscovery, EI Segundo, CA, USA). All probes in the analysis were used without preselection or filtering. We performed supervised comparative analysis to examine the statistical significance of differentially expressed genes based on the primary tumor status (pT). For this purpose, we compared the differential expression between high-stage (pT2-pT4) and low-stage (pTa-pT1) UCs to perform functional profiles focusing on those related to the heparan sulfate proteoglycan metabolic process (GO:0030201). Further survival analysis was performed in all cases from GES31684 by separating the cases into high-expression and low-expression clusters to computerize the prognostic impact of *SULF1* and *SULF2* genes.

### Patients and tumor specimens

This study was approved by the institutional review board (IRB10302015) of Chi Mei Medical Center and (KMUHIRB-E(I)-20160023) of Kaohsiung Medical University Hospital. We retrieved urothelial carcinoma cases diagnosed between 1996 and 2004 for immunohistochemical study and survival analysis. A total of 635 consecutively treated well-characterized urothelial carcinomas were enrolled, including 340 tumors originating from the UT and 295 arising from the UB. All patients were treated initially by surgical intervention with curative intent. As a rule, UBUC patients with pT3 or pT4 tumors or with nodal involvement received cisplatin-based adjuvant chemotherapy. However, only 29 of 106 pT3 or pT4 and nodal positive UTUC patients received cisplatin-based adjuvant chemotherapy. The criteria for clinicopathological evaluation were essentially identical to those used in our previous work [35, 36]. Two pathologists (PIL & CFL) re-evaluated the hematoxylin-eosin sections of all cases. For validation of *SULF1* transcript level, 24 UTUC and 24 UBUC snap frozen samples with high percentage (> 70%) of tumor components were retrieved.

### RNA retraction and quantitative real-time RT-PCR

We extracted total RNA from both cell lines and snap frozen tumor samples using the RNeasy Mini Kit (QIAGEN). The extracted total RNAs were subjected to reverse-transcription reactions using SuperScript III (Invitrogen) for cDNA synthesis. *SULF1* mRNA abundance levels were measured with pre-designed TaqMan assay (Hs00290918_m1) coupled with the ABI StepOnePlus System (Applied Biosystems). We calculated the fold level of expression of *SULF1* relative to normal adjacent tissues using a comparative Ct method, and *POLR2A* (Hs01108291_m1) was used as the internal control for normalization. The protocol was described previously [37, 38, 39].

### Cell culture

UC cell lines, including RT4, T24, TCCSUP and J82, were purchased from ATCC (Manassas, VA 20108, USA). TSGH8301, BFTC-905, and BFTC-909 cell lines were obtained from the Food Industry Research and Development Institute (Hsinchu, Taiwan). The RTCC1 UTUC cell line derived from the renal pelvis was sourced from Professor Lien-Chai Chiang at Kaohsiung Medical University [40]. These cells were cultured on the suggested medium using the conditions described in our previous work [37].

### Western blot

The tumor cells were lysed with cell lysis buffer containing 25 μg protein, separated by 4–12% gradient NuPAGE gel (Invitrogen, Carlsbad, CA) and transferred onto polyvinylidene difluoride membranes (Amersham, Bioscience, Buckinghamshire, UK). The membranes were probed with antibodies at 4°C overnight against SULF1 (1:1000, H-41, Santa Cruz), Akt (1:1000, Cell Signaling Technology), phosphorylated Akt^Ser473^ (1:1000, Cell Signaling Technology) and GAPDH as a loading control (6C5, 1:10,000, Millipore) after blocking with 5% skimmed milk in TBST buffer at room temperature for 1 hour. The secondary antibody was incubated at room temperature for 1.5 hours, and proteins were visualized by a chemiluminescence system (Amersham Biosciences).

### RNA interference

The lentiviral vectors obtained from Taiwan National RNAi Core Facility, including pLKO.1-*shLacZ* (TRCN0000072223: 5′-TGTTCGCATTAT CCGAACCAT-3′) and pLKO.1-*shSULF1* (TRCN0000 051098: 5′- CCCAAATATGAACGGGTCAAA -3′; TRCN 0000051100: 5′- CCAAGACCTAAGAATCTTGAT -3′), were used to set up stably SULF1-silenced clones of TSGH8301 and TCCSUP cell lines with the short-hairpin RNAs against SULF1 expression (*shSULF1*). These three vectors were transfected into HEK293 cells by Lipofectamine 2000 to create viruses, as previous described [37, 41].

### 2,3-bis-(2-methoxy-4-nitro-5-sulfophenyl)-2H-tetrazolium-5-carboxanilide (XTT)-based assay (Sigma, St. Louis, MO, USA)

The tumor cells were seeded into 96-well flat-bottom plates with phenol red-free medium at a density of 3000–5000 cells per well for 48 hours. The cells were incubated at 37°C in a humidified atmosphere containing 5% CO^2^. After 24, 48, or 72 hours of incubation, we removed the culture medium and added 20 μl of XTT reaction solution to each well and incubated the cultures for another 4 hours at 37°C. The optical density was measured with an enzyme-linked-immunosorbent assay (ELISA) microplate reader (GloMax Discover, Promega) for absorbance at a wavelength of 450 nm against a reference wavelength of 630 nm.

### Migration and invasion assays

The migration and invasion ability of cells were determined by the Boyden chamber technique (transwell analysis). The cell migration assay was performed with Falcon HTS FluoroBlok 24-well inserts (BD Biosciences). The 24-well Collagen-Based Cell Invasion Assay (Millipore) was used in the cell invasion assay. In brief, we rehydrated each insert by adding serum-free medium, replacing it with serum-free suspension with equal amounts of cells in the upper chamber, and incubating the cells for 12 to 24 hours to allow the cells to migrate toward/invade the lower chamber containing 10% FBS. After removing the non-invading cells in the upper chamber, the cells invading through the inserts were stained with the supplied dye, dissolved in extraction buffer, and transferred to 96-well plates for colorimetric reading at 560 nm by using ELISA microplate reader (GloMax Discover, Promega) [42, 43].

### Expression, small RNA interference plasmids, and transfection

Cysteines 87 and 88 of SULF1 (designated as SULF-1 delta CC) were mutated to alanines for blocking *N*-formylglycine modification of Cys87 of SULF-1 [44]. Transfected plasmids with wild type and mutant type SULF1 were cultured in BFTC909 cell lines for reexpression then analyzed using western blotting [44].

### Statistical analyses

Statistical analyses were performed using SPSS V.14.0 software (SPSS Inc. Chicago, IL, USA). The Chi-square test was performed to correlate SULF1 immunoexpression to various clinicopathological parameters. The end points analyzed were disease-specific survival (DSS) and metastasis-free survival (MeFS), as calculated from the starting date of curative operation to the date on which an event developed. Patients lost to follow-up were censored on the latest follow-up date. We plotted survival curves using the Kaplan-Meier method and the long-rank test to evaluate prognostic differences between groups. Parameters demonstrating less than 0.05 in the univariate analysis were subsequently enrolled in multivariate tests conducted using the Cox proportional hazards model. Student's *t*-test was used to analyze quantitative RT-PCR and functional assays for cell line samples. For all analyses, we used two-sided tests of significance with *P* < 0.05 considered significant.

## SUPPLEMENTARY MATERIALS FIGURES AND TABLES


