# Supplementary material for: Sulfatase-1 overexpression indicates poor prognosis in urothelial carcinoma of the urinary bladder and upper tract
Source: Oncotarget. 2017 May 3;8(29):47216–29. doi: 10.18632/oncotarget.17590 (PMC5564558; doi:10.18632/oncotarget.17590)
Supplement: Supplementary file 1 [file oncotarget-08-47216-s001.pdf]

Sulfatase-1 overexpression indicates poor prognosis in urothelial carcinoma of the urinary bladder and upper tract

Supplementary Materials

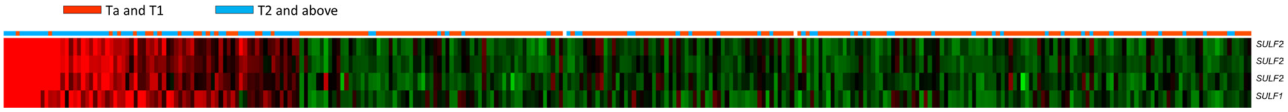

Supplementary Figure 1: Analysis of transcriptmoe database in urothelial carcinoma from GSE32894. Focusing on those involving heparan sulfate proteoglycan metabolic process revealed similar results to that from GSE31684. SULF1 remains to be the most significantly up-regulated gene associated with increments of pT status, followed by *SULF2*.

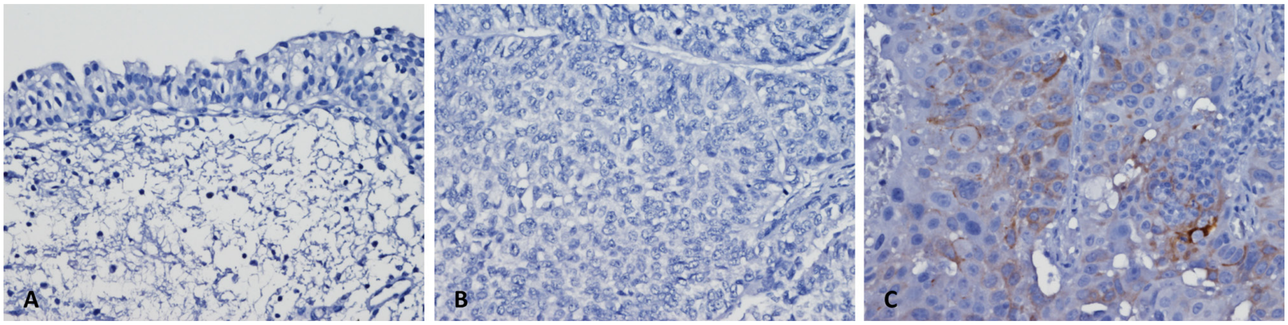

Supplementary Figure 2: Validation of SULF2 protein expression. Immunhistochemically, SULF2 is barely detected in non-invasive UC (**A**) and shows a mild increase in superficially invasive UC (**B**). The representative high-grade and high-stage UC shows a bright SULF2 immunoreactivity (**C**).

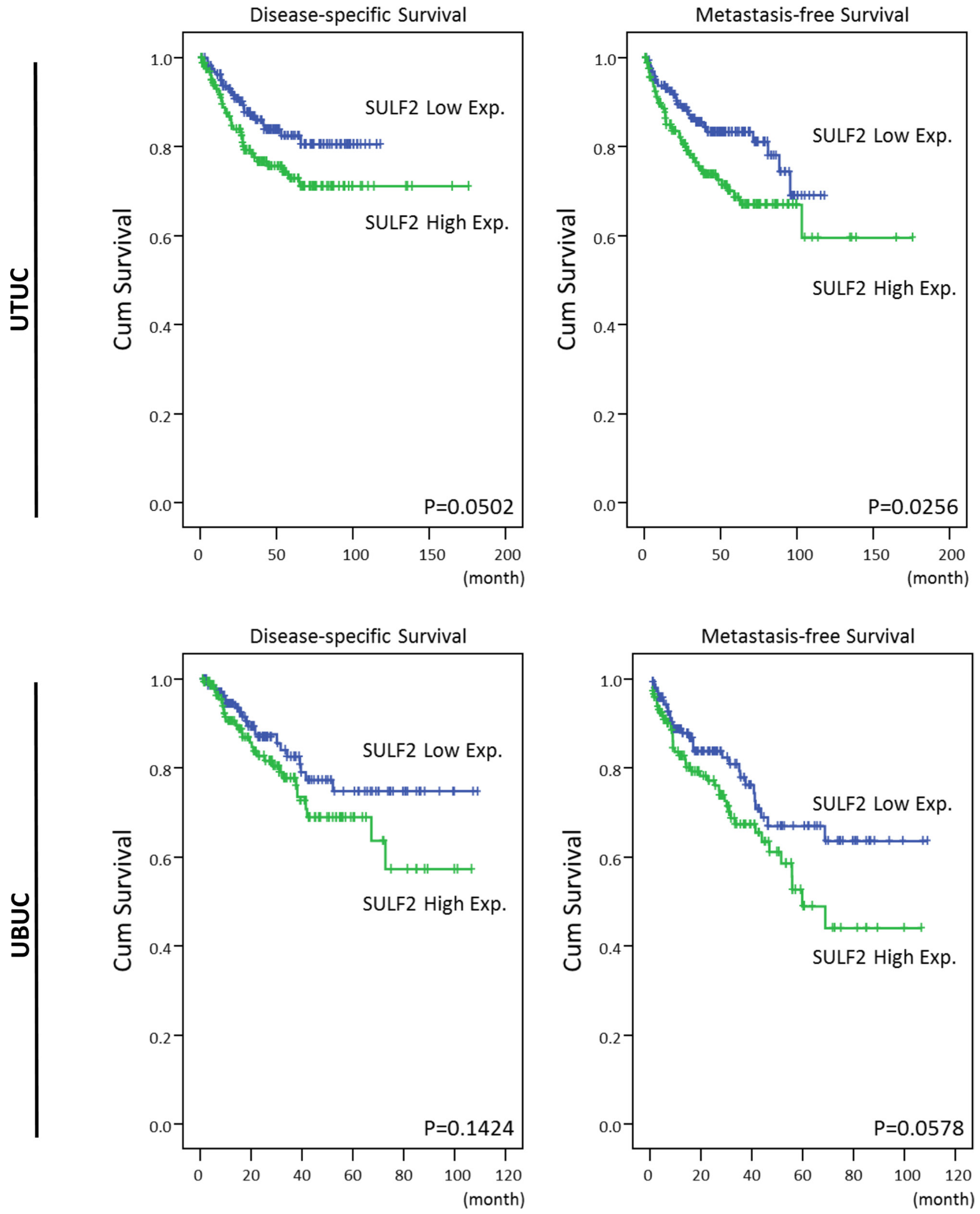

Supplementary Figure 3: Kaplan-Meier plots of disease-specific survival (DSS) and metastasis-free survival (MeFS) of UTUCs and UBUCs. SULF2 expression only significantly predicts inferior MeFS in UTUC (*P* = 0.0256).

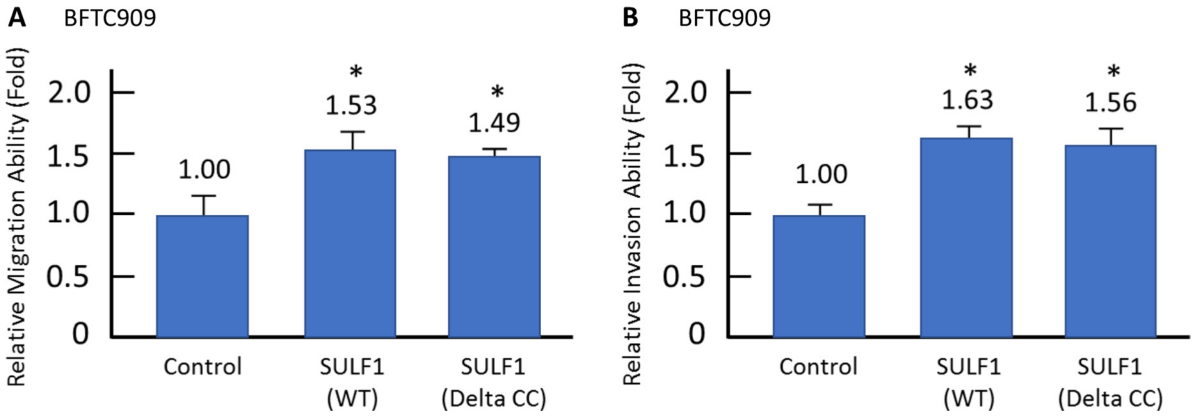

Supplementary Figure 4: SULF1 expression promotes cell migration and invasion. We confirmed SULF1 expression promotes cell migration and invasion with gain-of function of SULF1 (WT) in BFTC909 cell line. Interestingly, the effects could also be appreciated in those with delta CC-mutated SULF1, suggesting its oncogenic role dose not totally relay on the enzymatic function.

Supplemenetary Table 1: Summary of differentially expressed genes associated with heparan sulfate proteoglycan metabolic process in the transcriptome of urothelial carcinoma of urinary bladder (GSE32894)

Probe	Comparing T2-4 to Ta-T1		Gene Symbol	Biological Process	Molecular Function
	log ratio	*p*-value			
ILMN_1667460	1.036	0	SULF2	heparan sulfate proteoglycan metabolic process, metabolic process	arylsulfatase activity, calcium ion binding, hydrolase activity
ILMN_1686981	0.7979	0	SULF2	heparan sulfate proteoglycan metabolic process, metabolic process	arylsulfatase activity, calcium ion binding, hydrolase activity
ILMN_1702363	1.4253	0	SULF1	apoptosis, heparan sulfate proteoglycan metabolic process, metabolic process	arylsulfatase activity, calcium ion binding, hydrolase activity
ILMN_2345142	0.7303	0	SULF2	heparan sulfate proteoglycan metabolic process, metabolic process	arylsulfatase activity, calcium ion binding, hydrolase activity

Supplemenetary Table 2: Correlations between SULF2 expression and other important clinicopathological parameters in urothelial carcinomas

Parameter	Category	Upper Urinary Tract Urothelial Carcinoma				Urinary Bladder Urothelial Carcinoma			
		Case No.	SULF2 Expression		*p*-value	Case No.	SULF2 Expression		*p*-value
			Low	High			Low	High	
Gender	Male	158	76	82	0.514	216	106	110	0.667
	Female	182	94	88		79	41	38	
Age (years)	< 65	138	72	66	0.508	121	57	64	0.435
	≥ 65	202	98	104		174	90	84	
Tumor location	Renal pelvis	141	59	82	**0.020***	-	-	-	-
	Ureter	150	80	70		-	-	-	-
	Renal pelvis & ureter	49	31	18		-	-	-	-
Multifocality	Single	278	132	146	**0.049***	-	-	-	-
	Multifocal	62	38	24		-	-	-	-
Primary tumor (T)	Ta	89	72	17	**< 0.001***	84	58	26	**< 0.001***
	T1	92	59	33		88	48	40	
	T2-T4	159	39	120		123	41	82	
Nodal metastasis	Negative (N0)	312	164	148	**0.002***	266	144	122	**< 0.001***
	Positive (N1–N2)	28	6	22		29	3	26	
Histological grade	Low grade	56	41	15	**< 0.001***	56	39	17	**0.001***
	High grade	284	129	155		239	118	131	
Vascular invasion	Absent	234	133	101	**< 0.001***	246	128	118	0.090
	Present	106	37	69		49	19	30	
Perineural invasion	Absent	321	162	159	0.479	275	138	137	0.655
	Present	19	8	11		20	9	11	
Mitotic rate (per 10 high power fields)	< 10	173	99	74	0.007*	139	76	63	0.116
	> = 10	167	71	96		156	71	85	

*Statistically significant.

Supplemenetary Table 3: Univariate log-rank analysis for disease-specific and metastasis-free survivals in upper urinary tract and urinary bladder urothelial carcinoma

Parameter	Category	Case No.	Upper urinary tract urothelial carcinoma				Case No.	urinary bladder urothelial carcinoma			
			Disease-specific Survival		Metastasis-free Survival			Disease-specific Survival		Metastasis-free Survival	
			No. of event	*p*-value	No. of event	*p*-value		No. of event	*p*-value	No. of event	*p*-value
SULF2 expression	Low	170	24	0.0502	**27**	**0.0256***	147	21	0.1424	31	0.0578
	High	170	37		**43**		148	31		45	

* Statistically significant.
